# The role of XBP-1-mediated unfolded protein response in colorectal cancer progression-a regulatory mechanism associated with lncRNA-miRNA-mRNA network

**DOI:** 10.1186/s12935-021-02167-5

**Published:** 2021-09-14

**Authors:** Yong Wang, Jingyu Zhang, Shuang Zheng

**Affiliations:** 1 Department of Colorectal Surgery, The First Affiliated Hospital of Nanjing Medical University, Nanjing, 210029 Jiangsu China; 2 Department of Gastrointestinal Oncology, the Second People’s Hospital of Lianyungang, Lianyungang, 200023 Jiangsu China; 3 Department of General Surgery, Huangyan Hospital of Wenzhou Medical University, Taizhou First People’s Hospital, No. 218, Hengjie Road, Huangyan District, Taizhou City, 318020 Zhejiang Province China

**Keywords:** Colorectal cancer, XBP-1, Endoplasmic reticulum stress, KCNQ1OT1, XIST

## Abstract

**Background:**

We aim to identify the expression and analyze the molecular action of dysregulated lncRNA-miRNA mediated by XBP-1 in colorectal cancer (CRC).

**Methods:**

Here, we identified XBP-1-mediated dysregulated lncRNAs and miRNAs in CRC by bioinformatics analysis. The expression level of lncRNAs and miRNA was measured using quantitative real time PCR, and the expression of XBP-1, as well as apoptosis-related proteins, were detected by western blot. CCK-8 and TUNEL assays were performed to determine cell proliferation and apoptosis, respectively. Luciferase reporter assay was conducted to verify the binding relationship among lncRNA-miRNA-XBP-1. BALB/c nude mice were inoculated subcutaneously with HCT116 cells to establish tumor-bearing mice model. Histological analysis was carried out by HE staining and immunohistochemical staining.

**Results:**

Six downregulated lncRNAs (SLFNL1-AS1, KCNQ1OT1, NEAT1, XIST, AC016876.2, AC026362.1), four dysregulated miRNAs (miR-500a-3p, miR-370-3p, miR-2467-3p, miR-512-3p) and upregulated XBP-1 were identified in CRC cell lines. Gain- and loss-of-function experiments showed that overexpression of KCNQ1OT1/XIST promoted cell proliferation and suppressed cell apoptosis. In addition, overexpression of KCNQ1OT1/XIST partly abolished the inhibitory effects of XBP-1u knockdown or tunicamycin, an activator of endoplasmic reticulum stress, on CRC cell viability loss and apoptosis. Furthermore, KCNQ1OT1/XIST aggravated tumor growth *in vivo* by regulating endoplasmic reticulum stress and cell apoptosis.

**Conclusions:**

This study has constructed lncRNA-miRNA-mRNA networks based on XBP-1 in CRC, and disclosed the regulatory mechanism of action, providing a set of pivotal biomarkers for future molecular investigation and targeted treatment of CRC.

## Background

Colorectal cancer (CRC) is one of the most common malignant neoplasms worldwide, approximately accounting for 10 % of all diagnosed cancers and cancer related deaths annually worldwide [1]. In the past decades, great improvement of the diagnosis and treatment of CRC has been achieved; however, drug resistance, distant metastasis and recurrence still lead to a poor prognosis in CRC patients [2, 3]. Up to date, the precise mechanisms how CRC occurs and develops are still not clearly clarified. Recent studies on the biology of non-coding RNAs, especially long-noncoding RNAs (lncRNAs) and microRNAs (miRNAs), which serve crucial functions in numerous biological processes, have drawn intense interest in exploration of potential mechanism underlying cancer development. Numerous lncRNAs and miRNAs have been found to promote or suppress cancers, and been verified as promising diagnostic or prognostic biomarkers for CRC. Thus, based on lncRNA-miRNA, it is of significant importance to explore potential mechanisms of action and discover effective diagnosis biomarkers and therapeutic targets for CRC.

Endoplasmic reticulum stress (ERS) is a cellular adaptive mechanism that occurs in response to the disruption of ER homeostasis such as nutrient deprivation, hypoxia, and alterations in glycosylation status and calcium flux, which then triggers the unfolded protein response (UPR) [4]. Activation of UPR promotes protein folding and clearance of misfolded proteins by down-regulating protein translation and/or by pushing ER-associated degradation (ERAD) pathway [5, 6]. However, once ERS persists or is excessive, UPR activates cell death program and triggers cell death. In the past decades, increasing evidence has linked the UPR to cancer progression due to its ability of regulation of various cellular functions [7, 8].

X-box binding protein 1 (XBP-1) is an important transcription factor in the UPR pathway, which has been reported to accelerate tumor growth by inhibiting apoptosis [9]. In UPR process, inositol requirrung enzyme 1 (IRE1) oligomerizes and is activated by autophosphorylation to produce ribonuclease activity, which processes the mRNA encoding of XBP-1, splicing unsliced XBP-1 (XBP-1u) into spliced XBP-1 (XBP-1 s) by non-canonical splicing in cytoplasm [10]. XBP-1u has been widely considered as the precursor of XBP-1 s, and XBP-1 s plays a vital role in ER stress-induced UPR [11], while XBP-1u was regarded as the major form of XBP-1 under non-ER stress condition. XBP-1 s has been reported to be widely expressed in cancers, such as multiple myeloma, hepatocellular carcinoma, and CRC [12–14]. Particularly, XBP-1 s has been reported to promote CRC cell proliferation, acting as an oncogenic factor. However, recent studies have shown that XBP-1u also plays a unique function in regulating various biological pathways in cancers [15]. XBP-1u can stabilize the mouse double minute 2 (MDM2) protein, thereby promoting the reduction of the tumor suppressor gene p53 [16]. Besides, XBP-1u can inhibit cell autophagy by promoting the degradation of FOXO1 in cancer cells [17]. These evidences indicate that XBP-1 might be a crucial regulator of tumorigenesis; however, the specific role of XBP-1 s and XBP-1u has not been fully elucidated yet.

In this study, we focused on the role of XBP-1 in CRC from the perspective of XBP-1u and XBP-1 s, so as to clarify the influences of XBP-1-mediated UPR during the occurrence and development of CRC. Furthermore, biology of lncRNA-miRNA network based on XBP-1 mRNA was explored, and cellular bioactivities of relevant lncRNAs were conducted *in vitro* and *in vivo*, which provides novel promising diagnostic biomarkers and therapeutic targets for CRC.

## Materials and methods

### Cell culture

Normal cell line NCM460 and human CRC cell lines (SW620, SW480, HT-29 and HCT116) were purchased from Cell Bank of Chinese Academy of Sciences. All cells were cultured in DMEM (Thermo Fisher Scientific, Inc., Waltham, MA, USA) supplemented with 10 % fetal bovine serum (FBS; Thermo Fisher Scientific, Inc.) at 37 °C in a 5 % CO_2_ incubator.

### Quantitative real time PCR (qRT-PCR)

Total RNA was extracted using Trizol reagent (Invitrogen, Carlsbad, California, USA), quantified using NanoDrop, and reversely transcribed into complementary DNA (cDNA) using PrimeScript real-time reagent kit (Takara Bio, Japan) in accordance with the manufacturer’s instructions. After that, SYBR Select Master Mix (Applied Biosystems, Waltham, Massachusetts) was applied to carry out qRT-PCR on an ABI 7900 system (Applied Biosystems). Relative expression of the target gene was examined using 2^−△△Ct^ method and was normalized to β-actin.

### Western blot

Total protein was extracted using RIPA lysis buffer containing phenylmethylsulfonyl fluoride (PMSF; Beyotime Biotechnology, Shanghai, China) and quantified by BSA quantification assay in accordance with the manufacturer’s protocol. 30 µg of protein were separated by sodium dodecyl sulphate-polyacrylamide gel electrophoresis (SDS-PAGE) and transferred to polyvinylidene difluoride (PVDF) membranes (Millipore, Billerica, MA, USA). The membranes were blocked in the 5 % non-fat milk for 2 h at room temperature, followed by incubation with primary antibodies at 4 °C overnight and incubation with horseradish peroxidase-labeled secondary antibody for 2 h at room temperature on the next day. Finally, the membranes were exposed with enhanced chemiluminescence as described by the manufacturer (Beyotime Biotechnology).

### Cell transfection

The KCNQ1OT1 overexpression vector (Ov-KCNQ1OT1), XIST overexpression vector (Ov-XIST) and the negative control vector (Ov-NC) were synthetized and constructed by Ribocio (China). HCT116 cells were transfected with Ov-KCNQ1OT1/Ov-XIST or Ov-NC using Lipofectamine 2000 (Life Technologies, USA) following the manufacturer’s protocols.

### CCK-8 assay

Cell proliferation was determined using Cell Counting Kit-8 (Beyotime Biotechnology) in accordance with the manufacturer’s protocol. Briefly, cells were seeded in 96-well plates and grown at 37 °C for 12, 24 and 48 h, respectively. At the indicated time point, 10 µl of CCK-8 reagent was added to each well and cells were incubated for another 3 h. Finally, the absorbance at 450 nm was measured using a microplate reader (Bio-Rad, Hercules, CA, USA).

### TUNEL assay

Cell apoptosis was determined by terminal dexynucleotidyl transferase (TdT)-mediated dUTP nick and labeling (TUNEL) staining. Briefly, cells were fixed in 4 % paraformaldehyde and permeated with 1 % Triton-100. After washing with PBS twice, the cells were incubated with the TUNEL assay reagent (Roche Biochemicals, Mannheim, Germany) at 37 °C for 1 h. The fluorescein-labeled TUNEL-positive cells were captured under an inverted fluorescence microscope.

### Luciferase reporter assay

The luciferase-3′-UTR reporter constructs were generated by introducing the 3′-UTRs of KCNQ1OT1 carrying the putative miRNAs binding site into a luciferase reporter vector to generate KCNQ1OT1-WT or KCNQ1OT1-MUT. Then, miRNA mimic and the mimic control were co-transfected into HCT116 cells with KCNQ1OT1-WT or KCNQ1OT1-MUT. After transfection for 48 h, luciferase activities were measured using a Dual-Luciferase reporter assay kit (Promega, Madison, WI, USA).

### Animal experiments

Male BALB/c nude mice were housed in temperature- and humidity-controlled conditions on a 12-h light/dark cycle with free access to water and food. After acclimation for a week, all mice were randomly assigned into six groups (n = 6 per group). HCT116 cells overexpressing KCNQ1OT1 or XIST were inoculated subcutaneously into the flank of mice. Tumor growth was monitored every 3 days. Tumor volume was calculated as followed: volume = width^2^ × length/2. In addition, to investigate the role of KCNQ1OT1 or XIST against 5-fluorouracil (5-Fu) resistance, mice were received 5-Fu treatment (35 mg/kg; 2 times/week) when the tumor volume reached 50–100 mm^3^. 21 days later, all mice were euthanized by inhalation of 5 % isoflurane (Hairui Chemical; cat. no. HR135327) for 30 s and cervical dislocation. Subsequently, tumors were dissected, photographed, and weighted. All animal experiments were in accordance with the Guide for the Care and Use of Laboratory Animal (National Institutes of Health Publication), and were approved by the ethics committee of Taizhou First People’s Hospital.

### Histological and histochemical assays

The dissected tumors were fixed in 10 % paraformaldehyde for 24 h. Then, tissues were dehydrated in ethanol, embedded in paraffin and cut into sections with 4-µm thickness. These sections were stained with hematoxylin and eosin (H&E) to observe histological changes under an optical microscope. For histochemical assay, the sections were deparaffinized, rehydrated, and treated with 3 % hydrogen peroxide, followed by being blocked with 10 % goat serum at 37 °C for 30 min. After washing, the sections were incubated with anti-GRP78 antibody at 4 °C overnight. After that, the sections were incubated with secondary antibody at room temperature for 1 h, and then counterstained with hematoxylin for 30 s and visualized with DAB (ZSJQ, Beijing, China).

### Statistical analysis

The results were analyzed with GraphPad Prism 7.0 (GraphPad, La Jolla, CA, USA) and presented as means of the groups (± SD). To detect the differences, one-way ANOVA followed by Turkey’s post hoc analysis was performed. In all analyses, p < 0.05 was considered to be statistically significant.

## Results

### XBP-1-asssociated lncRNA-miRNA-mRNA network

**Fig. 1:**
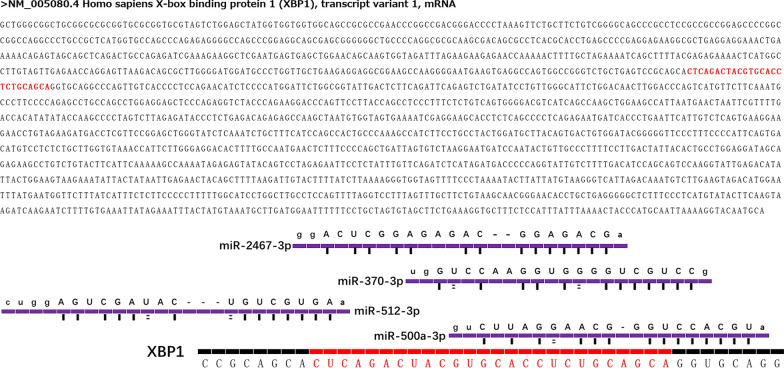
Interaction network by bioinformatic analysis. By searching NCBI and starbase website, four miNRAs (miR-500a-3p, miR-370-3p, miR-2467-3p and miR-512-3p) were found to have potential binding sites with the shear fragment of XBP-1 mRNA

**Fig. 2:**
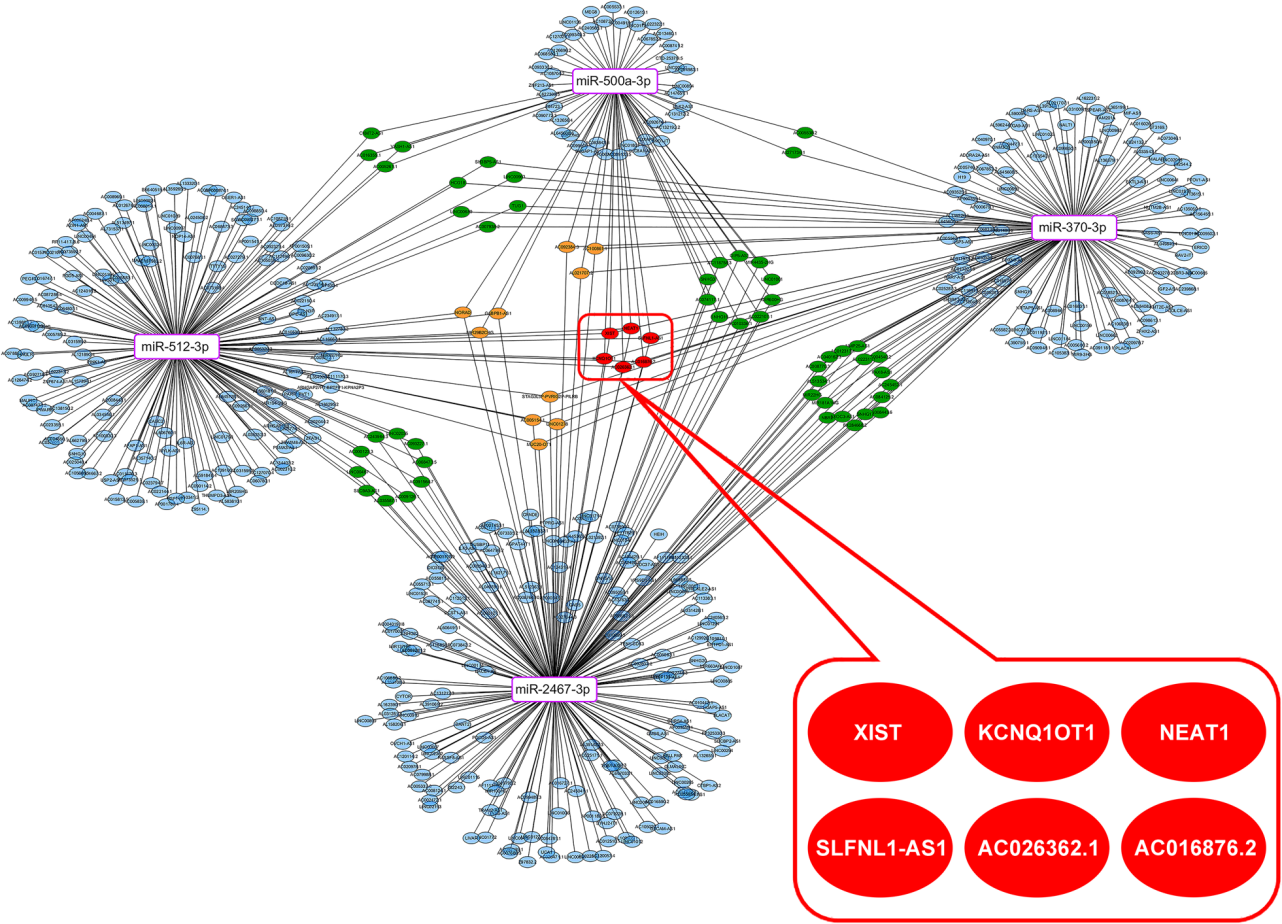
Interaction network by bioinformatic analysis. The lncRNA (SLFNL1-AS1, KCNQ1OT1, NEAT1, XIST, AC016876.2, AC026362.)-miRNA (miR-500a-3p, miR-370-3p, miR-2467-3p and miR-512-3p)-mRNA network. Red ellipses represent lncRNA, purple rectangles represent miRNA, and blue, orange and green ellipses represent mRNA

To investigate the specific role of XBP-1 in CRC, starBase analysis was conducted and many miRNAs that have potential binding relationships with XBP-1u were found. Among these miRNAs, there were only 4 miRNAs whose binding domains were located in the cut fragments, including miR-500a-3p, miR-370-3p, miR-2467-3p and miR-512-3p (Fig. 1). Interestingly, except miR-500a-3p, the other 3 miRNAs have been reported to inhibit the malignant phenotype of tumors [18–20]. Then, further upstream analysis was conducted and SLFNL1-AS1, KCNQ1OT1, NEAT1, XIST, AC016876.2 and AC026362.1 were lncRNAs that could bind to the above 4 miRNAs and be degraded. As shown in Fig. 2, the network revealed a preliminary connection between SLFNL1-AS1, KCNQ1OT1, NEAT1, XIST, AC016876.2, AC026362.1 and miR-500a-3p, miR-370-3p, miR-2467-3p, miR-512-3p.

### Expression level of lncRNA-miRNA-XBP-1 in CRC cell lines

**Fig. 3:**
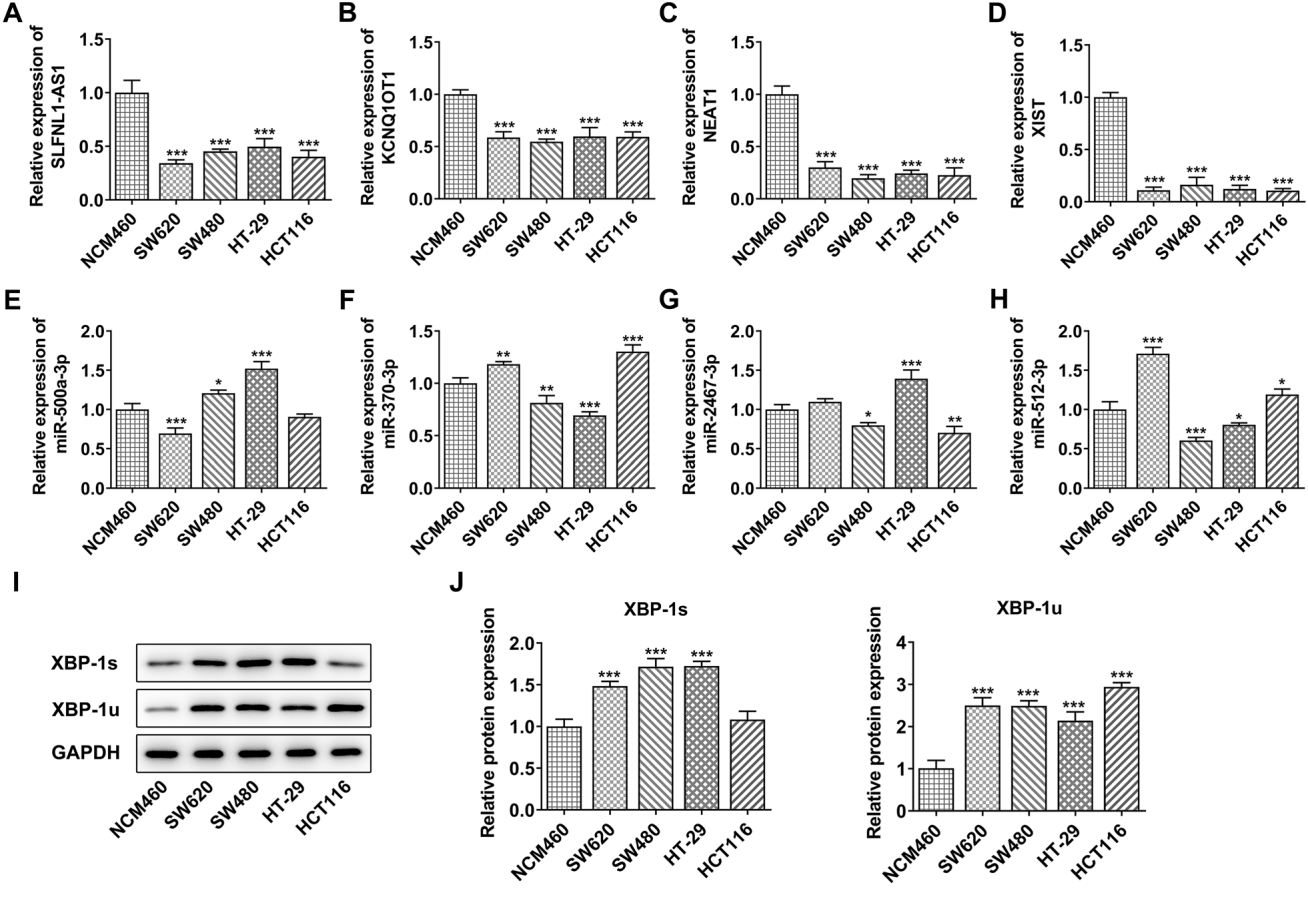
Expression level of lncRNA-miRNA-XBP-1 in CRC cell lines. **A**–**D** The expression level of lncRNAs (SLFNL1-AS1, KCNQ1OT1, NEAT1 and XIST) in normal cell line NCM460 and human CRC cell lines (SW620, SW480, HT-29 and HCT116) was detected using qRT-PCR. **E**–**H** The different expression levels of miRNAs (miR-500a-3p, miR-370-3p, miR-2467-3p and miR-512-3p) in NCM460 and human CRC cell lines was detected using qRT-PCR. **I**, **J** The protein expression of XBP-1 s and XBP-1u was measured using western blot. *, **, ***p < 0.05, 0.01, 0.001 vs. NCM460

Here, SLFNL1-AS1, KCNQ1OT1, NEAT1 and XIST were selected for investigation due to the poor information on AC016876.2 and AC026362.1. The different expression levels of lncRNAs, miRNAs and XBP-1 were measured in NCM460 and human CRC cell lines (SW620, SW480, HT-29 and HCT116). As shown in Fig. 3A–D, these expression levels of SLFNL1-AS1, KCNQ1OT1, NEAT1 and XIST were significantly down-regulated in CRC cell line, compared to those in NCM460. The expression level of these miRNAs had a slight decrease trend from the overall multiple relationship in CRC cell lines, but there was no obvious rule and difference between a certain cell line and a certain miRNA (Fig. 3E–H). Furthermore, the protein expression of XBP-1 s and XBP-1u were detected by western blot, and the results showed that the the overall expression of XBP-1u and XBP-1 s in CRC cell lines was higher than that in NCM460 (Fig. 3I, J).

### KCNQ1OT1/XIST overexpression induced ER stress and suppressed cell apoptosis

**Fig. 4:**
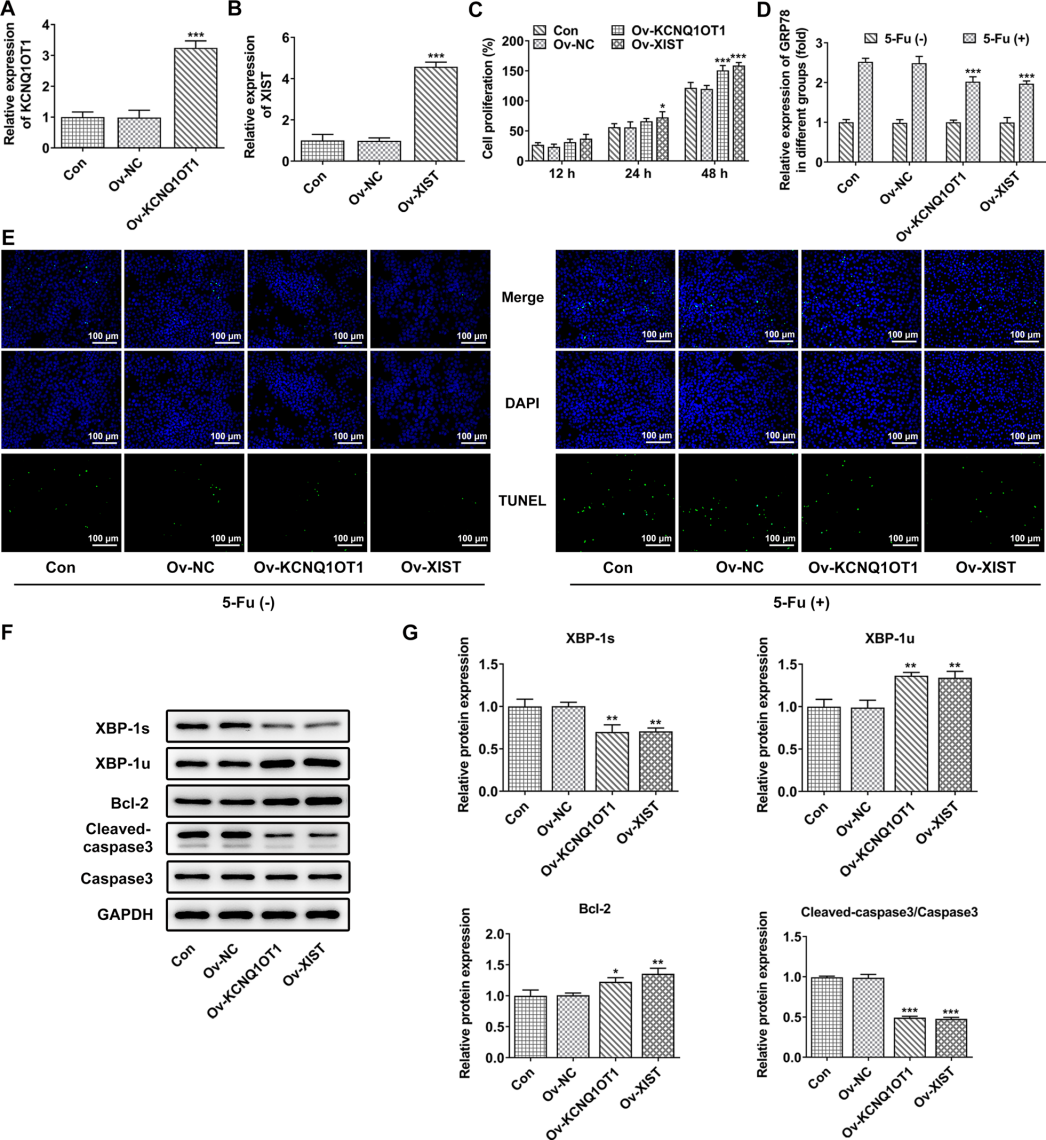
KCNQ1OT1/XIST overexpression induced ER stress and suppressed cell apoptosis. **A** Ov-KCNQ1OT1 and Ov-NC were transfected into HCT116, and the expression level of KCNQ1OT1 was detected using qRT-PCR. **B** Ov-XIST and Ov-NC were transfected into HCT116, and the expression level of XIST was detected using qRT-PCR. **C** CCK-8 assay was conducted to analyze cell proliferation ability **D** After transfection, HCT116 cells were treated with or without 5-Fu, and the expression of GRP78 was detected using qRT-PCR. **E** TUNEL assay was conducted to determine cell apoptosis. **F**, **G **The protein expression of XBP-1 s, XBP-1u, Bcl-2, cleaved caspase3 and capase3 were detected using western blot. *, **, ***p < 0.05, 0.01 and 0.001 vs. Ov NC

It has been verified that expression levels of SLFNL1-AS1, KCNQ1OT1, NEAT1 and XIST were down-regulated in CRC cell lines, which indicated the potential involvement of these lncRNAs in CRC progression. Here, KCNQ1OT1 and XIST were selected for further investigation. Then, Ov-KCNQ1OT1 and Ov-NC were transfected into HCT116, and the expression level of KCNQ1OT1 was significantly elevated upon Ov-KCNQ1OT1 transfection (Fig. 4A). Similarly, the expression level of XIST was also elevated upon Ov-XIST transfection in HCT116 cells (Fig. 4B). CCK-8 assay showed that either KCNQ1OT1 or XIST overexpression significantly promoted cell proliferation ability (Fig. 4C). Glucose-regulated protein 78 (GRP78, also called Bip/HSPA5), characterized as an ER chaperone, is a master modulator of UPR. After transfection, HCT116 cells were treated with or without 5-Fu. As shown in Fig. 4D, overexpression of KCNQ1OT1 or XIST did not influence the level of GRP78. 5-Fu treatment increased the level of GRP78 of HCT116 cells, which was partly abolished by overexpression of KCNQ1OT1 or XIST. Further Tunel assay revealed that 5-Fu promoted cell apoptosis, which was partly hindered by overexpression of KCNQ1OT1 or XIST (Fig. 4E). Besides, KCNQ1OT1 or XIST overexpression also had an inhibitory effect on cell apoptosis, which was further verified by decreased protein expression of cleaved caspase3 and increased protein expression of Bcl-2 upon transfection. Moreover, overexpression of KCNQ1OT1 or XIST obviously decreased the protein expression of XBP-1 s but increased the protein expression of XBP-1u (Fig. 4F, G).

### ER stress-induced apoptosis was partly inhibited by KCNQ1OT1/XIST

**Fig. 5:**
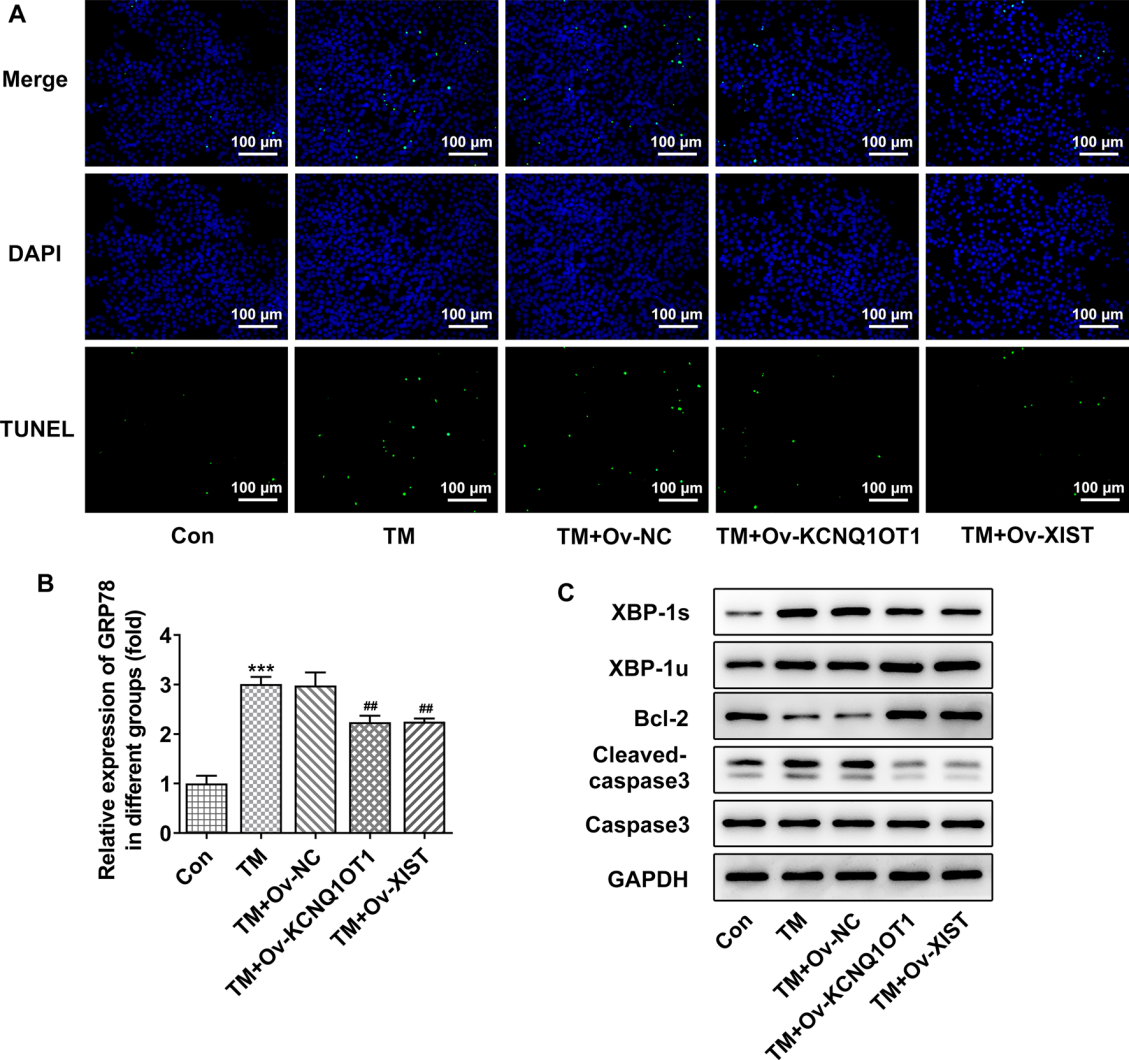
ER stress-induced apoptosis was partly inhibited by KCNQ1OT1/XIST. **A** HCT116 cells were treated with tunicamycin (TM), an activator of ER stress, and transfected with Ov-KCNQ1OT1 or Ov-XIST. TUNEL assay was conducted to determine cell apoptosis. **B** The expression level of GRP78 was detected by qRT-PCR. **C** The protein expression of XBP-1 s, XBP-1u, Bcl-2, cleaved caspase3 and capase3 were detected using western blot. ***p < 0.001 vs. TM; ##p < 0.01 vs. TM + Ov NC

To ensure the role of ER stress underlying the biological regulation of lncRNAs, HCT116 cells were treated with tunicamycin (TM), an activator of ER stress. As shown in Fig. 5A, TM significantly increased TUNEL-positive cells, indicating that ER stress promoted cell apoptosis, which was partly abolished by KCNQ1OT1 or XIST. Besides, western blot assay revealed that TM reduced Bcl-2 expression and elevated cleaved caspase3, and these alternations were reversed by KCNQ1OT1 or XIST (Fig. 5B, C), consistent with the results from TUNEL assay results. Furthermore, TM increased both XBP-1 s and XBP-1u, indicating that ER stress induced a high level of XBP-1; however, the level of XBP-1 s was then inhibited by KCNQ1OT1 or XIST, but the level of XBP-1u was led to a higher degree. Moreover, the elevated level of GRP78 induced by TM was hindered by KCNQ1OT1 or XIST. The results above suggested that activation of ER stress, along with a higher expression of XBP-1u and GRP78, promoted cell apoptosis, and this pro-apoptotic effect was weakened by KCNQ1OT1 or XIST.

### The regulatory role of XBP-1u in KCNQ1OT1/XIST associated cell proliferation and apoptosis

**Fig. 6:**
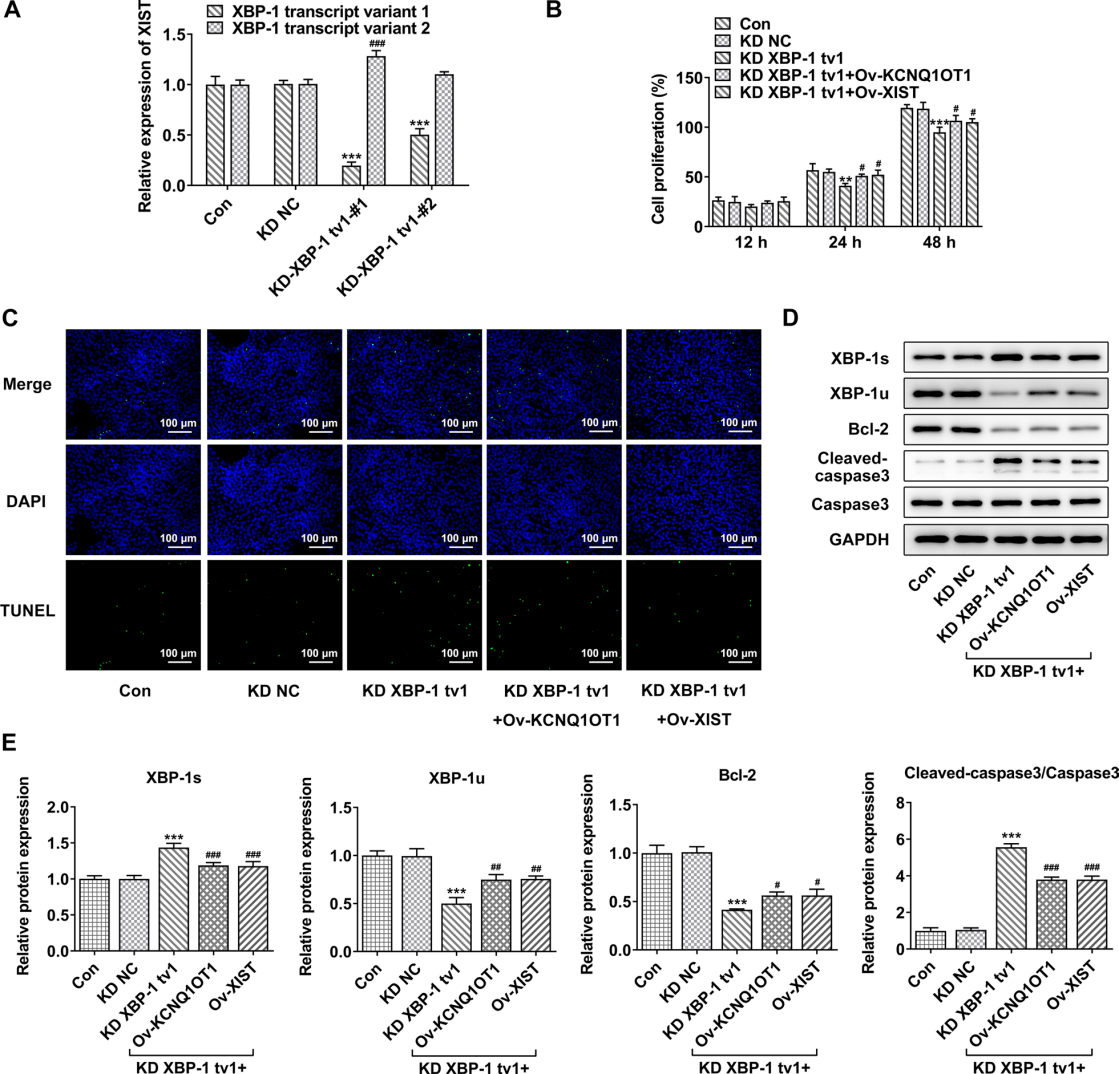
The regulatory role of XBP-1u in KCNQ1OT1/XIST associated cell proliferation and apoptosis. **A** HCT116 cells were transfected with XBP-1u knockdown vector (KD-XBP-1 tv1#1/2) and the negative control (KD NC). The expression level of XBP-1 transcript variant 1 (tv1) and XBP-1 tv2. **B** HCT116 cells were transfected with KD-XBP-1 tv1 or co-transfected with KD-XBP-1 tv1 and KCNQ1OT1/XIST. CCK-8 assay was performed to detected cell proliferation ability. **C** TUNEL assay was conducted to detect cell apoptosis. **D**, **E** western blot assay was performed to measure the protein expression of XBP-1 s, XBP-1u, Bcl-2, cleaved caspase3 and caspase3. **, ***p < 0.01, 0.001 vs. KD NC; #, ##, ###p < 0.05, 0.01, 0.001 vs. KD XBP-1 tv1

Next, to further verify the role of XBP-1u underlying the biological behavior of KCNQ1OT1/XIST during CRC progression, HCT116 cells were transfected with XBP-1u knockdown vector (KD-XBP-1 tv1#1/2) and the negative control (KD NC). After transfection, mRNA level of XBP-1 tv1 was significantly declined, especially in KD-XBP-1 tv1#1 group, thus, KD-XBP-1 tv1#1 was used for the further experiments (Fig. 6A). Then, HCT116 cells were transfected with KD-XBP-1 tv1 or co-transfected with KD-XBP-1 tv1 and KCNQ1OT1/XIST. CCK-8 assay showed that knockdown of XBP-1 tv1 decreased cell proliferation ability, which was abolished by KCNQ1OT1/XIST (Fig. 6B). Similarly, TUNEL assay revealed an elevated cell apoptosis rate upon XBP-1 tv1, which was partly reversed by KCNQ1OT1 or XIST (Fig. 6C). Besides, the decreased XBP-1u and Bcl-2 and the increased cleaved caspase3 induced by XBP-1 tv1 knockdown were impaired by KCNQ1OT1or XIST (Fig. 6D, E).

### The directly targeted miRNAs of XBP-1 and KCNQ1OT1/XIST

#### The binding interaction between miRNAs and lncRNAs or XBP-1u

**Fig. 7:**
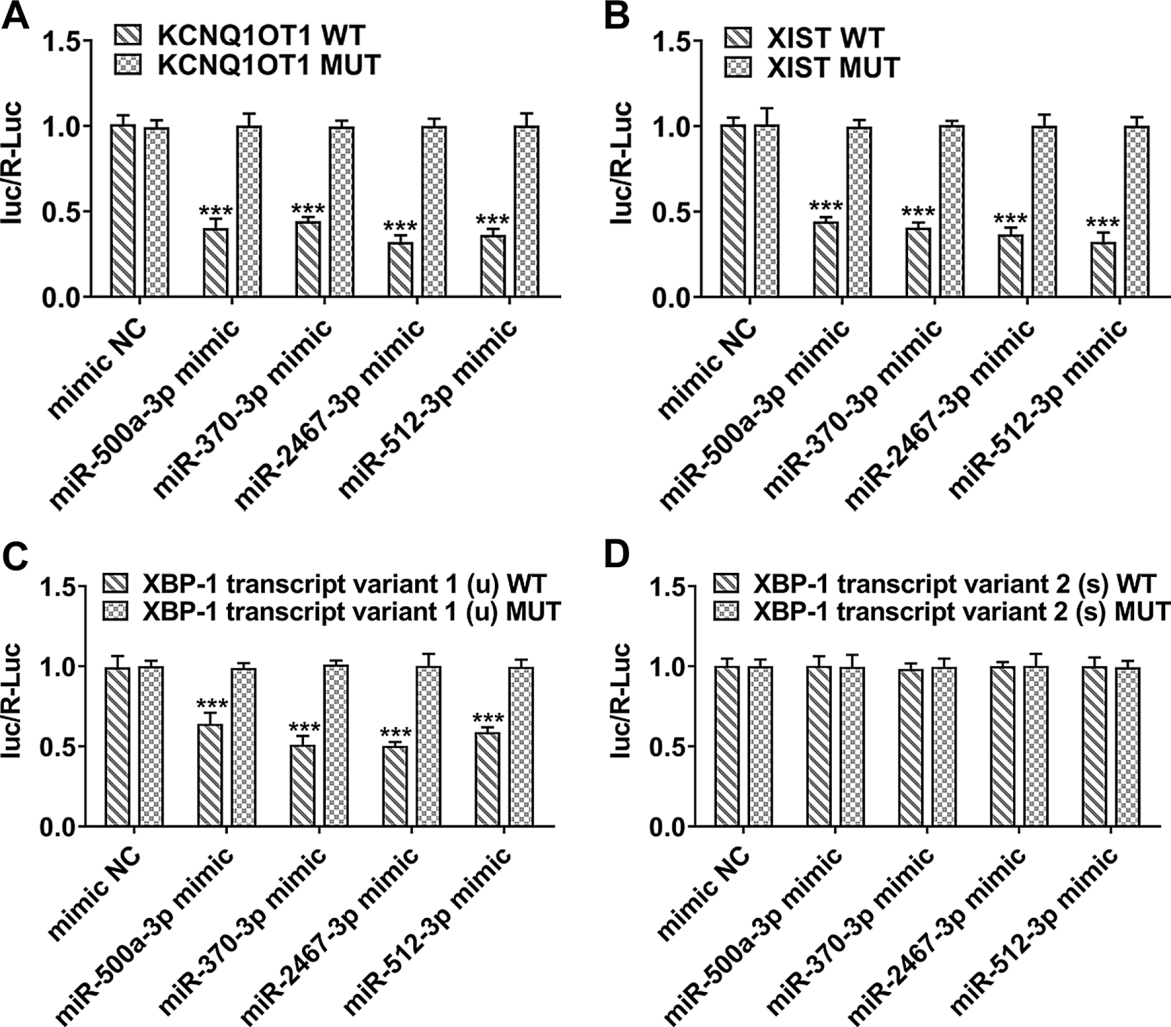
The directly targeted miRNAs of XBP-1 and KCNQ1OT1/XIST. **A** HCT116 cells were co-transfected with KCNQ1OT1 WT/KCNQ1OT1 MUT and miR-500a-3p/miR-370-3p/miR-2467-3p/miR-512-3p mimic, and the relative luciferase activity was determined using luciferase reporter assay. ***p<0.001 vs KCNQ1OT1 WT. **B** HCT116 cells were co-transfected with XIST WT/XIST MUT and miR-500a-3p/miR-370-3p/miR-2467-3p/miR-512-3p mimic, and the relative luciferase activity was determined using luciferase reporter assay. ***p < 0.001 vs XIST WT. **C** HCT116 cells were co-transfected with XBP-1u WT/ XBP-1u MUT and miR-500a-3p/miR-370-3p/miR-2467-3p/miR-512-3p mimic, and the relative luciferase activity was determined using luciferase reporter assay. ***p < 0.001 vs XBP-1u WT. **D** HCT116 cells were co-transfected with XBP-1s WT/ XBP-1s MUT and miR-500a-3p/miR-370-3p/miR-2467-3p/miR-512-3p mimic, and the relative luciferase activity was determined using luciferase reporter assay

As known from StarBase, miRNAs (miR-500a-3p, miR-370-3p, miR-2467-3p, miR-512-3p) have potential binding relationships with XBP-1u and lncRNAs (KCNQ1OT1, XIST), which were then verified using luciferase reporter assay. The luciferase activity was remarkably decreased in HCT116 cells co-transfected with KCNQ1OT1 WT and miR-500a-3p/miR-370-3p/miR-2467-3p/miR-512-3p mimic (Fig. 7A), indicating that these miRNAs could directly bind to 3’UTR of KCNQ1OT1. Similarly, these miRNAs could directly bind to 3’UTR of XIST (Fig. 7B). Furthermore, luciferase reporter assay showed that co-transfection with XBP-tv1 WT and miRNAs mimic exhibited an obvious decrease of luciferase activity, whereas the luciferase activity did not change when cells were co-transfected with XBP-tv2 WT and miRNAs mimic (Fig. 7C, D), indicating that these miRNAs could directly bind to XBP-1 tv1 but not bind to XBP-1 tv2. In other words, miR-500a-3p, miR-370-3p, miR-2467-3p and miR-512-3p could directly target XBP-1u but not XBP-1s.

### KCNQ1OT1/XIST overexpression aggravated tumor growth in vivo

**Fig. 8:**
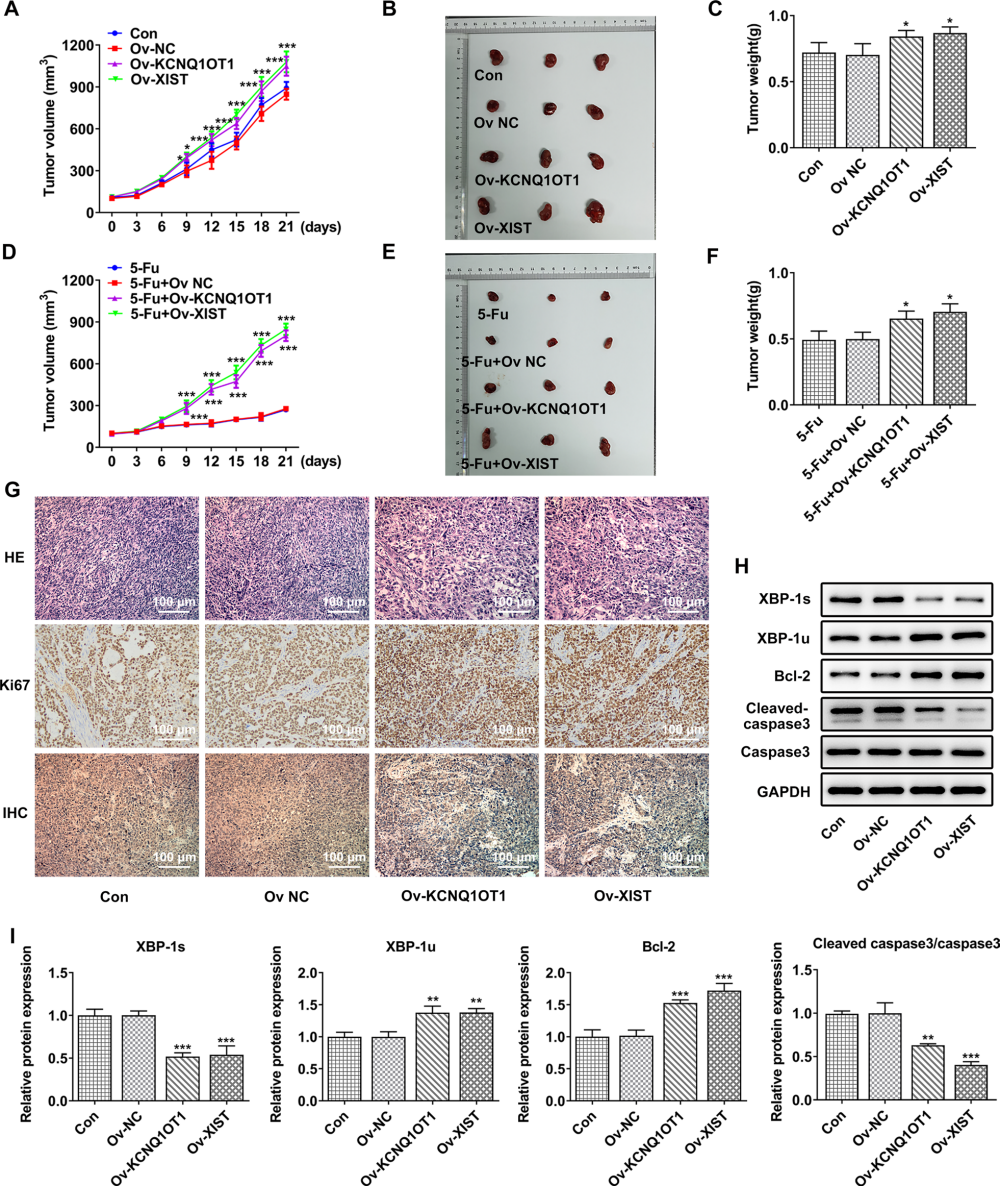
KCNQ1OT1/XIST overexpression aggravated tumor growth in vivo. **A**–**C** Mice were inoculated subcutaneously with HCT116 cells overexpressing KCNQ1OT1 or XIST. The tumor volume was recorded. After sacrifice, the tumor size was observed and tumor weight was recorded. **D**–**F** Mice were inoculated subcutaneously with HCT116 cells overexpressing KCNQ1OT1 or XIST and treated with 5-Fu. The tumor volume was recorded. After sacrifice, the tumor size was observed and tumor weight was recorded. **G** HE staining was performed to evaluate histological change, and IHC staining was performed to assess Ki67 and GRP78 expression in tumor. **H**, **I** western blot assay was performed to measure the protein expression of XBP-1s, XBP-1u, Bcl-2, cleaved caspase3 and caspase3. *, **, ***p < 0.05, 0.01, 0.001 vs Ov NC or 5-FU+Ov NC

To further verify the role of KCNQ1OT1/XIST in CRC, mice were inoculated subcutaneously with HCT116 cells overexpressing KCNQ1OT1 or XIST. As shown in Fig. 8A–C, either the tumor weight or tumor size was enlarged in mice overexpressing KCNQ1OT1 or XIST, indicating that KCNQ1OT1/XIST overexpression induced tumor growth in mice with colorectal cancer. In addition, the enlarged tumor weight and tumor size were also observed in mice received 5-Fu treatment and overexpression of KCNQ1OT1 or XIST, compared to that only received 5-Fu treatment (Fig. 8D–F), indicating that KCNQ1OT1/XIST overexpression weakened the anti-tumor effect of 5-Fu in vivo and strengthened the drug resistance to 5-Fu in colorectal cancer. Histological analysis further verified the tumor-promoting effect of KCNQ1OT1/XIST overexpression (Fig. 8G). In addition, IHC staining exhibited that the Ki67 (a marker of proliferation) was upregulated while GRP78 was down-regulated by KCNQ1OT1/XIST (Fig. 8G), suggesting that KCNQ1OT1/XIST promoted cancer cell proliferation but inhibited ERS activation. Moreover, western blot assay exhibited that KCNQ1OT1/XIST overexpression greatly reduced the protein expression of XBP-1s and cleaved caspase3 and elevated the protein expression of XBP-1u and Bcl-2 (Fig. 8H, I).

## Discussion

CRC is one of the common malignant tumors of the digestive tract, and its high prevalence rate has become a public health problem worldwide [21]. Nowadays, although great improvements of therapies have been achieved to resist CRC, the curative effects on patients are still limited [22, 23]; thus, discovering more novel biomarkers and elaborating more precise molecular mechanism underlying the regulation of tumorigenesis and development is of great importance.

In recent years, it is well-established that targeting the ERS response is an effective manner for inhibiting the tumor growth of various cancer [24–26], therefore more and more concentration has been focused on determining how to utilizing the ERS response or URP as an approach for treating cancers, as well as CRC. For example, Aloe-emodin served as a candidate in the treatment of CRC through upregulating URP proteins and inducing ERS-dependent apoptosis [23]; TSPYL5 suppressed CRC cell proliferation, migration and invasion via activation of ERS, indicating that targeting the ERS response using TSPYL5 might be a promising strategy for CRC treatment [27]. XBP-1, an important branch of the URP during ERS activation, has been regarded as a crucial target for anti-cancer strategies, as genetic inhibition of XBP-1 resulted in diminished tumor growth, and several compound, obtained from a small molecule screen for inhibitors of XBP-1 activation, exhibited potential anti-tumor activity [28]. In particular, XBP-1 was expressed at high levels in metastatic tumor samples of patients with CRC, and *in vitro* studies revealed that overexpression of XBP-1 promoted CRC cell invasion, while inhibition of XBP-1 significantly suppressed cell invasion [29]. Based on these findings, this study further investigate the role of XBP-1(especially its splicing form: XBP-1 s and XBP-1u)-mediated URP in CRC progression. It is observed that both of XBP-1 s and XBP-1u were expressed at high levels in CRC cell lines, compared to the normal cell line. From bioinformatics analysis, KCNQ1OT1 and XIST, expressing at low levels in CRC cell line, were identified to indirectly targeting XBP-1u. To the best of our knowledge, only a previous study reported that KCNQ1OT1 could regulate ERS by directly targeting GRP78, thus affecting cerebral ischemia-reperfusion injury [30], and the effects of KCNQ1OT1/XIST on ERS in cancers have not previously investigated. Here, we observed that KCNQ1OT1/XIST overexpression greatly downregulated XBP-1 s but upregulated XBP-1u. Gain- and loss-of-function experiments exhibited that overexpression of KCNQ1OT1/XIST weakened ERS activation-induced cell apoptosis, and partly abolished XBP-1u knockdown-caused cell viability loss and cell apoptosis, suggesting that targeting XBP-1-mediated ERS response using KCNQ1OT1 or XIST might be a promising strategy for CRC treatment.

A large body of evidence have implicated that lncRNAs, a class of regulatory RNA, play a key role in regulating development and growth of a tumor. lncRNAs act as ceRNAs which sponge miRNAs through miRNA response elements (MREs), thus regulating the expression of targeted mRNAs, thereby contributing to various pathological processes and influencing cancer progression. Increasing studies have well evidenced that lncRNA-miRNA-mRNA ceRNA network plays vital roles in the tumorigenesis and progression of CRC [30–32]. For example, a study by Zhuang et al. determined that lncRNA MALAT1 upregulated the SLAIN2 by sponging miR-106b-5p, thus enhancing the microtubules mobility and promoting the invasion and metastasis of CRC [33]; Xu et al. found that lncRNA SNHG6 was upregulated in CRC tissues and cells, and high expression of SNHG6 promoted CRC cell growth and metastasis by acting as a molecular sponge of miR-26a/b to regulate their common target EZH2 [34]. Chen et al. observed that lncRNA-ZFAS1 could bind to miR-150-5p and inhibit VEGFA degradation, thus contributing to the development of CRC [35]. In the present study, we constructed a unique lncRNA-miRNA-mRNA network based on the ceRNA hypothesis and the important role of XBP-1 in tumorigenesis. We initially predicted the miRNAs of XBP-1 based on starBase analysis. According to the literature research, we identified 4 key miRNAs (miR-500a-3p, miR-370-3p, miR-2467-3p, miR-512-3p), based on which, we further identified 4 downregulated lncRNAs (SLFNL1-AS1, KCNQ1OT1, NEAT1, XIST), indicating a potential involvement of these lncRNAs in CRC progression. As we known, lncRNAs, as the ceRNA of miRNA, can compete with mRNA for miRNA, thus reducing the content of free miRNA, so as to realize the regulation of targeted mRNA. Meanwhile, as lncRNAs can bind to miRNA, it also acts as a target gene for miRNA, which reduces the stability of lncRNA and promotes its degradation, which might explain why the expression level of these lncRNAs were downregulated in CRC cell lines while there was no significant difference in the expression level of these miRNAs in CRC cell lines. A series of cellular behaviors experiments revealed that overexpression of KCNQ1OT1 or XIST greatly aggravated CRC cell proliferation *in vitro* and tumor growth *in vivo*, acting as oncogene in CRC. Mechanistically, these miRNAs (miR-500a-3p, miR-370-3p, miR-2467-3p, miR-512-3p) have multiple binding sites with KCNQ1OT1/XIST and XBP-1u, indicating that KCNQ1OT1/XIST might influence the expression of XBP-1u by competitively binding to these miRNAs, contributing to the progression of CRC.

However, some limitations still exist in the present study. Firstly, we only investigated cell proliferation and cell apoptosis to evaluate the effect of KCNQ1OT1/XIST on cellular behaviors. Actually, other cellular behaviors, such as cell migration, invasion, epithelial-mesenchymal transition, as well as cell cycle progression, are also deserved to be explored to enrich the role of this molecular axis in CRC. Secondly, the signaling transduction of ERS underlying XBP-1-mediated lncRNA-miRNA network in CRC remains unclear. Finally, the present study only pointed out a part of the XBP-1-regulated genes in CRC, discovering more potential XBP-1-regulated genes is also deserved to be investigated to extend the regulatory role of XBP-1 in CRC. We will proceed with these problems in our future work.

## Conclusions

To sum up, our study disclosed an interesting relationship underlying XBP-1-mediated lncRNA-miRNA network in CRC. We found that KCNQ1OT1 or XIST act as the sponge of miRNAs (miR-500a-3p, miR-370-3p, miR-2467-3p, miR-512-3p), accounting for the regulatory role of XBP-1-mediated ERS response in CRC tumorigenesis and development. This study elucidated novel molecular mechanisms involved in initiation and progression of CRC, providing promising clues for clinical diagnosis and therapy.

## Data Availability

All data generated or analyzed during this study are included in this published article.
